# Stakeholder Views on the Idea of Medicines Reuse in the UK

**DOI:** 10.3390/pharmacy9020085

**Published:** 2021-04-16

**Authors:** Parastou Donyai, Rachel McCrindle, Terence K. L. Hui, R. Simon Sherratt

**Affiliations:** 1 Reading School of Pharmacy, University of Reading, P.O. Box 226, Whiteknights, Reading RG6 6AP, UK; 2 Department of Biomedical Engineering, School of Biological Sciences, University of Reading, Whiteknights, Reading RG6 6AY, UK; r.j.mccrindle@reading.ac.uk (R.M.); t.hui@reading.ac.uk (T.K.L.H.); r.s.sherratt@reading.ac.uk (R.S.S.)

**Keywords:** medicines, reuse, recycle, medicines reuse, attitudes

## Abstract

People’s views about medicines reuse are being examined in a handful of qualitative studies and this commentary adds to that work by drawing on our own discussions with groups of stakeholders in the UK in the past two years. The reuse of medicines within the community pharmacy setting is not permitted in the UK but our multidisciplinary team anticipates that this position will change in the coming years as medication shortages and worries about environmental waste and financial losses from the destruction of unused medicines are brought to the fore. Indeed, for many stakeholders, the issue of waste is a strong feature of conversations about medicines reuse. In addition to this, stakeholders identify the numerous barriers to medicines reuse in the UK. This includes the current uncertainty about the quality of unused medicines returned to pharmacies, which could otherwise be reused. However, stakeholders have also been very willing to propose solutions to a range of existing barriers. Our commentary draws on stakeholder meetings to elaborate the range of views about medicines reuse within a UK context. The challenge is to move forward from these views to advance the technologies that will facilitate medicines reuse practically as well as legally.

## 1. Introduction

A limited number of studies have examined people’s views about the concept of ‘medicines reuse’ [1,2,3,4] and the purpose of this commentary is to add to this body of knowledge by reporting on our own discussions about the topic with groups of stakeholders in the UK in the past 24 months.

The concept of ‘medicines reuse’ itself is open to different interpretations and definitions. For example, some might understand it to relate to reusing a patient’s own medicines when they are admitted to a hospital ward [5]; or the concept might be related to the recycling of medicinal components or packaging in future manufacturing processes [6]. The phrase medicines reuse has also been used to refer to the repurposing of old drugs for new conditions. A number of related terminologies also exist, including re-dispense, recycle, redistribute and reverse flow. These ideas and related concepts, although important, are not the focus of the current paper. Here, we use the term ‘medicines reuse’ to mean the idea that within a community pharmacy context, “medication returned by one patient can be dispensed by a pharmacist to another patient (instead of disposal as waste–which is what currently takes place)”. Our paper is focused within a UK context, where pharmacists working within community pharmacies are not permitted to reuse medicines. What prevents medicines reuse in this setting is a combination of the law, professional guidance and past precedence.

In the UK, apparently how a medicine is sourced is not generally relevant as long as a medicine is supplied in accordance with the relevant prescription, for the specific purposes of part 12 of the Human Medicines Regulation 2012 [7]. However, reusing medicines reportedly invalidates the terms and conditions under which medicines are supplied from wholesalers [8]. Additionally, because legislation governing the supply of medicines requires persons trading medicines (other than directly supplying to patients) to hold a wholesale dealer’s license [unless supplies are small, take place occasionally, are not-for-profit and not for onward wholesale distribution], this legislation also limits the receipt and redistribution of returned medicines between different units/legal entities along a supply chain (e.g., from one pharmacy to another) [9]. Furthermore, under normal circumstances, medicines reuse is not recommended by the Department of Health because the quality of any medicine that has left the pharmacy cannot be guaranteed [10,11]. In fact, in the UK, none of the regulatory and professional bodies currently support the reuse of medicines within the community pharmacy setting. This includes the Medicines and Healthcare Products Regulatory Agency (MHRA), the Association of British Pharmaceutical Industries (ABPI), the General Pharmaceutical Council (GPhC), the National Health Service (NHS), the British Medical Association (BMA), and the Royal Pharmaceutical Society (RPS). Our research group, however, is investigating the sustainability of this position.

Based on existing knowledge about pharmacy and the technology that might be integrated within pharmaceutical packaging, our multidisciplinary team Reuse of Medicines through Informatics, Networks and Sensors (ReMINDS) (www.reading.ac.uk/ReMINDS, accessed on 14 April 2020) is composed of academics from the pharmacy, computer science and biomedical engineering fields at the University of Reading. ‘ReMINDS’ communicates our opinion on medicines reuse candidly (we are pro researching medicines-reuse) so our paper is arranged as a commentary rather than a research paper, the aim being to present new viewpoints on an existing problem, while also drawing on original data. Here, we draw on key themes conveyed to our group by a range of stakeholders in meetings organized to discuss medicines reuse, while acknowledging that our paper is imbued with personal opinion, in line with Berterö’s definition of a commentary [12].

We draw on our discussions with a range of stakeholders that includes young people, future pharmacists, pharmacists working within the primary-care, community, hospital, and homecare settings, pharmaceutical industry representatives, specialists in medicines supply, patients, and researchers.

## 2. The Wastage of Medicines

Medicines reuse as a concept stems from the problem of medication waste. After all, if there was zero medication wastage, there would be no medicines *to* reuse. Thus, a range of ideas about medicines reuse are expressed by people, directly in relation to the creation and prevention of medication waste.

A range of practices and settings are thought to contribute to medication waste. For example, using multi-compartment compliance aids (MCCAs) as a practice is thought to be wasteful; in MCCAs, individual doses, having been removed from their packaging, are placed with other medicines within distinct compartments a month or more in advance of actual consumption, rendering the medicines ‘expended’ as soon as the MCCA is prepared. Then if a patient’s medication regimen is changed (e.g., a medication is discontinued), the entire content of their MCCA becomes unusable for that patient—this is because it is too risky to remove individual discontinued tablets from the compartments (risk of error) so the entire contents have to be discarded once the regimen changes. MCCAs are utilized widely within care homes, where there is also a notion by some stakeholders that care-home staff contribute to the wastage of medicines by excessive reordering and the stockpiling of medicines.

Outside of formal care settings, another factor associated with medication waste is medication non-adherence, where patients fail to fully follow the dosage instructions of their medication, for example by not taking their medicines at all or failing to complete the full course. The reasons for non-adherence are complex, multi-factorial and well researched but some noteworthy insights from our patient stakeholders include the need to create conditions that allow patients to disclose their real medication-taking patterns, and to address their fear, disinterest or lack of understanding about medicines, some of which are potentially engendered, it seems, through the physical appearance (design/text) of medicinal packaging.

An additional behaviour recognized and discussed by our stakeholders is the unnecessary/over-ordering of medication, for example patients ordering large pack sizes, and stockpiling medication, or over/inappropriate prescribing by doctors. This issue is especially important in the UK since patients are sometimes seen not to be ‘accountable’ for behaviours such as over-ordering, because many do not directly pay for their medication and bear none of the financial costs. There is arguably then no real barrier to intentional or unintentional medication stockpiling, with some of the people we have spoken to suggesting that notifying patients about the monetary cost (albeit to the NHS) of their medicines (e.g., printing the price on the medicinal packet) might incentivize more responsible re-ordering behaviours—or that in any case alerting patients of their responsibility to reduce the NHS medicines spend is a worthwhile activity.

Doctors, pharmacists and other health professionals are also considered key actors who can reduce medicines waste by ‘taking responsibility’ for more sustainable practices. For example, by checking, discussing and challenging quantities and medicines being re-ordered, completing medication reviews to ensure rational prescribing, prescribing lower quantities for more expensive medicines or medicines that are new to patients, and ensuring better stock control (including liaising with wholesalers and delivery companies) to avoid accumulating short-dated items (including on hospital wards) or ending up with medicines that are kept at the wrong storage temperature. Related to this is the notion of deprescribing (stopping superfluous medicines), which some patients and doctors avoid out of fear (of therapeutic repercussions). Finally, as patients’ own drugs (PODs) can still be used if they are admitted to hospital, another challenge is to ensure patients take their current medicines to hospital, to avoid duplicate dispensing, especially important where people have multiple admissions to hospital within a short period of time. Of course, this is not to deny that some PODs are judged to be of insufficient quality by hospital staff and re-dispensed in any case.

## 3. Barriers to Medicines Reuse

Similar to that reported elsewhere, our stakeholders had some concerns about medicines reuse. This included questions about the quality and safety of returned medicines and how these might be checked for suitability; and whether patients and consumers really store their medicines correctly at home, especially medicines requiring cold storage—and how people might be educated to do so. Our stakeholders recognized the potential for errors or contamination to occur within the supply chain. Further, they wondered about the cleanliness and potential for contamination of returned medication packs.

One of the concerns about medicines reuse relates to the practicality of operating such a scheme in UK community pharmacies. Community pharmacists have limited time for additional services, meaning that the addition of a medicines reuse scheme would doubtless require effective resourcing and incentivization. It also necessitates additional guidance and standard operating procedures for the receipt, separate storage, quality-assurance, and supply of reusable medicines within relatively small pharmacy spaces. National pharmacy bodies, for example, would be expected to publish consensus guidelines on medicines reuse. Or the NHS might consider taking back returned medication for storage and re-distribution. An additional challenge within community pharmacies relates to the reimbursement of prescription costs and the audit trail needed to prevent duplicate payments to pharmacies, while ensuring pharmacies are paid for the cost of administering medicines reuse.

There is also an expectation for medicines reuse to be financially logical, with implementation costs having to balance against the cost of the original medication. For example, any additional technology that might track the medicine’s storage conditions, batch number, manufacturing date/product age, expiry date, etc., to manage resupply, should reasonably be cost-effective. Stakeholders also highlight the potential paradox of having to add to a medicine’s carbon footprint in order to reduce its waste—the environmental harm of medicines reuse (e.g., via any additions to the packaging) must thus balance against that created by the medicine’s potential wastage if unused.

Two points came up specifically when we spoke to younger people about medicines reuse. Some conceptualized reuse as the process of taking back medicines and re-extracting the constituent elements. For packaged medicines, they thought that these are already routinely sent to other (poorer) countries for their use, but this practice is discouraged by the World Health Organisation which sees it as operating double standards.

Stakeholders pose other relevant questions within the UK context, including whether multiple re-use (re-dispensing) of a medicine might be permitted, and how recall of medicines might affect re-dispensed medicines. Another important issue is how current legislation to hold a wholesale dealer’s licence impacts on medicines reuse across different sections of the NHS.

A related matter concerns the falsified medicines directive (FMD), which describes a set of measures introduced in the European Union for the regulation of medicines trade, to prevent the appearance of fake medicines in the legal supply chain. February 2019 saw the implementation of two specific safety features on medicines determined by FMD; a unique identifier (a 2D data matrix code with product code, serial number, batch number, expiry date) on medicinal packaging scannable at fixed points along the supply chain, and tamper evident features (anti-tampering devices) on the pack. Thus, medicines can be verified in their movement through the supply chain, and ‘decommissioned’ at the final point, on supply to the patient. The stakeholders we engaged with duly ask how medicines then might be placed back within the supply chain in light of FMD, and how the safety features determined by FMD might be harvested to verify medicines for reuse. However, FMD will no longer apply in the UK following its exit from the European Union in 2021, and while this negates the need to ‘re-commission’ a medicine, the absence of safety features that might prevent falsified medicines from entering the supply chain is a less constructive step for medicines reuse.

## 4. Towards Solutions

Medicines reuse is not currently permitted in the UK but there have been ample questions and ideas from our stakeholders on how to promote engagement with such a scheme in the future. Some questions are, for example, how people might be incentivized to return their unused medicines to pharmacies in the first place, especially within the shelf life of the product. And how they might be educated to store their medication correctly at home to start with. How the stigma around returning medicines to pharmacies might be reduced—after all, these are medicines that would have been ordered/accepted but then left unused. Further, is there a need to take consent before supplying ‘reused’ medicines? How might we tackle negative perceptions about receiving what some might consider to be ‘second-hand medicines’, such that medicines reuse becomes socially acceptable, or indeed an obligation in light of eco-friendly movements? Perhaps in the future there might even be a system where people actively ‘opt out’ of receiving medicines within a reuse scheme.

Suggestions for changing popular opinion and social norms include teaching about the importance of medicines reuse, communicating success stories, and reshaping misconceptions about ‘re-used’ medicines. Stakeholders believe incentivizing uptake, or at least quantifying the overall benefits of medicines reuse could encourage engagement. For example, reusing medicines to prevent medicines shortages is a logical aim. Patients also want clearer information about the current fate of medicines returned to pharmacies. Other suggestions are to learn from existing groups such as ‘free cycle’, and to train pharmacists and other staff to promote engagement with medicines reuse, and indeed sustainable pharmacy more broadly, and engaging with popular media, celebrity advocates and social media influencers. The use of social media and technology (e.g., smartphones) is seen as plausible, indeed inevitable but patients also express that any developments in this area should be inclusive of older people, who use medicines the most, and poorer patients who might lack access to the newest smartphones.

The support of a range of official bodies too is seen as important for sanctioning medicines reuse in the first place, none of which currently approve medicines reuse within the community pharmacy setting. For example, engaging with medicines reuse might become a part of the community pharmacy contract and embedded within the pharmacy professional standards. Or administering medicines reuse might formally become one of the responsibilities of support staff within a pharmacy. Stakeholders suggest drawing on the experience of similar schemes in Greece [13] and the US [14] to overcome existing barriers to medicines reuse, and aiming to make medicines reuse as acceptable as reusing coffee cups and plastic bags.

Pharmaceutical companies are seen to play a key role and perhaps there could even be tax breaks for companies proactively changing their practices to facilitate reuse; or other incentives in lieu of their social responsibility. For example, pharmaceutical companies are recognized for holding primary raw data relating to the stability of medicines under different storage conditions—gaining access to these data might enable researchers to model and predict the integrity of medication stored in different home environments, to help define medication reuse criteria. Pharmaceutical companies might also explore whether *medication packaging* could be modified to colour-code sensitivity to environmental conditions, increase tolerance to these conditions, accommodate time/temperature indicators or other technology within the surface, or become more sustainable in itself. They might extend the usable shelf life of medicines. They might invest in the development of a secure supply chain for the return of medicines, sustainable technology that monitors medicine integrity during storage and use, or indeed tracks its whereabouts, and provide assurances about the safety, quality and cleanliness of returned medicines. Such technology would need to be secure and ensure the privacy, and even liberty of users. It would also need to be mindful of the primary users of medicines—for example, to prevent creating alarm if a visual quality indicator shows a potentially ‘invalidated’ medicine during first use.

Hospital pharmacies are recognized for their policies on medicines reuse (for using PODs), and pharmacies in general have risk management tools, which stakeholders expressed would be useful to learn from.

In terms of legislation, our stakeholders recognized the importance of engaging with the various professional and regulatory bodies to enable medicines reuse, recognizing the time and effort that would be required. Activities might include lobbying the MHRA for an exemption that would allow medicines to be reused within the community pharmacy setting; or illustrating potential cost savings to the Department of Health, at least for high-cost items or where drugs are vulnerable to shortages.

## 5. Discussion

Our stakeholders’ ideas about the wastage of medicines can be summarized as relating to practices around MCCAs, especially in care homes, medication non-adherence, over-ordering, over-prescribing, improper stock control and inadequate use of PODs. Their concerns about medicines reuse relate to the quality and safety of returned medicines, pharmacy resources and incentivization to deal with the process, the cost-benefits of such a scheme, and legislative barriers. Finally, their proposed solutions centre on educating and incentivizing the public, removing stigma around returning and reusing medicines, defining consent processes, using technology and social media, engaging with official bodies and the pharmaceutical industry, learning from existing practices in hospitals and lobbying regulators to change the law.

The issues identified as contributing to waste have long been recognized by others and in fact mirror many of the findings of a seminal report on the scale, causes and costs of waste medicines published in 2011 [15]. Indeed, the problem of medication waste is one of the main reasons for debating medicines reuse. This is alongside the high cost of medication, for example expensive cancer drugs in developed countries [16] and the cost of a range of other drugs for chronic and communicable diseases in developing countries such as India [17]. A small pilot in Singapore has also identified the huge potential for medicines reuse to reduce medication wastage and costs [18]. Researchers examining the benefit of long-term donation programmes in Europe, Africa and Latin America, against WHO’s formal advice to withhold such donations, also report a decrease in expenditure by both patients and health facilities [19]. The issue of lack of accountability for the over-ordering of medicines identified by our stakeholders associated with free prescriptions has also been debated before, with one suggestion being to charge a nominal £1 fee for prescription items to create a symbolic contract for patients to take their medicines more responsibly [20].

Some of the concerns relating to medicines reuse expressed by our stakeholders, as well as their proposed solutions, also feature in the limited number of studies that have systematically examined medication reuse. For example, Bekker and colleagues who examined views about medicines reuse in The Netherlands in 2014/15 identified two central requirements for the re-dispensing of returned medicines; namely, patient willingness to use and trust re-dispensed medicines and guaranteed product quality of re-dispensed medicines [2]. System requirements in that study were identified as relating to legal feasibility, financial aspects that should be taken into account and the roles stakeholders can fulfil [2]. Interestingly, in 2014 Liou and colleagues devised a quality control programme to ensure the safe recycling of metered dose inhalers within a hospital setting, focusing on microbial decontamination of the partially used devices [21]. When McRae and colleagues interviewed pharmacists in the UK about medicines reuse in 2014, they identified a range of criteria to be met for pharmacists to potentially accept the redistribution of tablet and capsule medication: “protection for pharmacists; guidance from the professional regulator; tamper evident seals; ‘as new’ packaging; technologies to indicate inappropriate storage and public engagement” [22]. Our own findings from interviews with members of the public in the UK exploring medicines reuse beliefs in 2016 was structured around the theory of planned behaviour [3]. We reported views on the potential economic and environmental benefits of medicines reuse alongside people’s worries about medication stability and safety. Our participants then also wondered if pharmacists had the time and storage space to dedicate to medicines reuse. The physical characteristics of reused medicines, and quality assurance and logistics of reuse processes were also seen to enable/obstruct engagement in medicines reuse [3]. Thus, our stakeholders’ views outlined here appear to chime with the concerns expressed by others in the past, and appear to be valid and reasonable to address.

While the number of studies in this field are limited, it is also clear that once people are consulted, there is an appetite for exploring how to make medicines reuse safe, and a limited number of ideas on how to do so in practice. One of the ideas that appears to be unique to our own exploration of views here is to engage pharmaceutical companies in sharing their raw stability data to be programmed into a system for monitoring the impact of storage conditions on the continued stability of medication stored in a patient’s home. We also found it interesting that when talking to our younger stakeholders, they imagined medicines reuse was already taking place, albeit at least via donations to developing countries. What is important about our work is that is brings together the views of a range of participants and reflects the latest thinking in this area. However, it is also interesting to note that as far back as 2007, Mackridge and Marriott spotted the potential that by using “modern packaging techniques, including tamper-evident seals and ‘smart’ labels that react to temperature and humidity, it would be possible to identify inappropriately stored medicines” [23].

## 6. What Next

Alongside stakeholder consultations described here and elsewhere [3], we have been progressing some of the practical ideas relating to medicines reuse within our multidisciplinary ReMINDS team. A review of the literature has allowed us to suggest a novel ReMINDS ecosystem as a solution for reusing returned prescribed medicines [24]. This system relies on active sensing technologies integrated with the Internet of Things platform to validate the quality and safety of medicines while interconnecting the relevant stakeholders. Additionally, we have developed the prototype for a novel digital time, temperature and humidity indicator using smart sensors with cloud connectivity as the key technology for verifying and enabling the reuse of returned medicines [25]. The past year has also seen a global pandemic impacting on the supply of medicines which in the UK has resulted in the temporary approval of medicines reuse within the hospice and care home sectors [26]. Our challenge now is to learn from the reuse of medicines within these settings and continue to explore the technological ways in which medicines reuse can be further progressed.
